# Absence of *Human Papillomavirus *in Benign and Malignant Breast Tissue

**DOI:** 10.30699/ijp.2019.89684.1847

**Published:** 2019-09-22

**Authors:** Maryam Kazemi Aghdam, Seyed Alireza Nadji, Azadeh Alvandimanesh, Maliheh Maliheh, Yassaman Khademi

**Affiliations:** 1 *Pediatric Pathology Research Center, Research Institute for Children’s Heath, Shahid Beheshti University of Medical Sciences, Tehran, Iran*; 2 *Department of Pathology, School of Medicine, Shahid Beheshti University of Medical Sciences, Tehran, Iran*; 3 *Virology Research Center (VRC), National Research Institute of Tuberculosis and Lung Diseases (NRITLD), Shahid Beheshti University of Medical Sciences, Tehran, Iran*; 4 *Department of Pathology, Shafa Hospital, Qazvin University of Medical Sciences, Qazvin, Iran*; 5 *Department of Pathology, Pathobiology Laboratory Center, Tehran, Iran*

**Keywords:** Breast, Benign, Malignant, Human Papillomavirus, Polymerase Chain reaction

## Abstract

**Background & Objective:**

Malignant breast tumors, which are one of the most important deadly cancers in women, like many other cancers, are proposed to be related to viruses etiologically. Proper management of breast carcinoma necessitates an identification of the etiological factors. *Human Papillomavirus* is considered to have an etiological role in breast carcinoma. We carried out this study to find out if *Human Papillomavirus*-DNA is present in the malignant and benign breast tissue in our patients.

**Methods:**

Seventy-five paraffin-embedded breast cancer tissues and 75 normal breast tissues and benign breast lesions were examined in this study (case-control) to look for *Human Papillomavirus*-DNA employing Nested Polymerase Chain reaction. The tissues were examined over a period of ten years in the pathology department of the Pathobiology Laboratory Center of Tehran.

**Results:**

No *Human Papillomavirus*-DNA was found in any of the malignant or control group specimens.

**Conclusion:**

Our results showed no evidence of Human Papillomavirus in cancerous and benign tissues, which is consistent with some other studies in English medical literature. More investigations using more specimens from different parts of the country are required to confirm the presence or absence of any connection between Human Papillomavirus and development of breast carcinoma in Iran.

## Introduction

Recently, researchers are concentrating on trials to prevent virus-related malignancies, which could prove to be hard but not unfeasible. Vaccination programs are expected to save many lives, not only by preventing viral infections, but also by preventing virus-associated cancers such as *hepatitis B *or *Human Papillomavirus *(HPV) (1, 2). On the other hand, it is suggested that for curing virus-related cancers, a multi-dimensional attack can be used, such as combination of a vaccine with a drug that prevents cancer cells to hide from the immune system (2).

About twenty percent of cancers in humans are thought to be caused by oncogenic viruses (3). The cancers that are highly suggested to be caused by DNA virus are hepatocellular carcinoma, lymphoma, nasopharyngeal carcinoma, cervical cancer, and Langerhans cell histiocytosis (3-5). Since the development of breast carcinoma is a multi-stage process, DNA infection may have a role in some stages (3, 4). Polymerase Chain reaction (PCR) technique has detected *Cytomegalovirus *(*CMV*), *Herpes simplex virus*-1 (*HSV*), *Epstein Barr virus* (*EBV*), HPV, and *Human herpes virus* type 8 (*HHV*-8) and *Mouse Mammary Tumor virus *(*MMTV*) in breast malignancies (3, 6, 7). However, the results are controversial (4, 8). 

Some researchers believe that the genotype of HPV determines its pathogenicity and the high-risk (HR) HPV16, 18 and 33 are to be blamed for about 70% of breast carcinomas in the world (4, 7). In cancerous cases, HR-HPV incidence was reported to be 47% in the United Kingdom, with the most common types being 39, 18, 45, 16, 35, and 59 (6). However, in India, the most commonly reported HPV types in malignant lesions were 16, 18 and 33 (9).

The carcinogenic effect of HPV is due to E6/ E7 oncoprotein expression, which increases the proliferation of cells, directing to more genomic unsteadiness and hindering apoptosis. Viral protein E2 normally controls the expression of these oncoproteins, but is frequently cancelled because of viral incorporation into the host gene through E2 section. E6 and E7 are also controlled by RNA splicing, and the long control region containing diverse binding sites for transcription factors together with the different number of viruses are controlled by the P97 promoter and enhancer activity (9).

If the role of viruses in the induction of breast cancer could be proved, there is an opportunity to prevent it (8). We conducted this research to identify the HPV DNA in tumoral breast tissue and illustrate the existence of any connection of HPV with breast carcinoma in Iran.

## Materials and Methods

**Patients and Controls**

The paraffin-embedded tissues of 75 patients who were diagnosed with breast carcinoma by one of the authors during a period of 10 years at the Pathobiology Laboratory Center in Tehran, Iran, are used in this study. The cases were diagnosed according to decisive factors in pathology textbooks. Sufficient tissue samples were taken for DNA extraction after re-examination of the slides under the light microscope. Small tissue samples were excluded (criterion of inclusion). All patients were Iranian, and the mean age was 48.2 years. Seventy five tissue samples were selected from the same department, which included fat necrosis, ductal ectasia, fibrocystic change (FCC), FCC and focal adenomatoid change, FCC and sclerosing adenosis, and reduction mastectomy specimens (years 2005 to 2014). The criterion for inclusion was negativity for malignancy.

The patients whose samples were used were anonymous. The data taken from the pathology reports is untouched. The University Ethics Committee code was: SBMU.REC.1393, 193.

**Tissue Preparation and Genome Extraction**

5-10 µm-thick-sections were cut from archive-biopsy blocks using a microtome and each section was collected into a sterile tube. The tissue sections were deparaffinized and rehydrated by xylene and alcohol and then lysed by tissue lysis buffer and Proteinase K. Genome extraction was conducted using RTP® DNA/ RNA Virus Mini Kit (Stratec Molecular GmbH, Berlin, Germany). The extracted DNA was frozen at -20°C. To assess the contamination during genome extraction, PCR-grade double distilled water was employed as a negative control in every single round of the procedure.

**Polymerase Chain Reaction (PCR)**

B-globin gene PCR amplification was applied for observing false-negative result and DNA adequacy, utilizing the PC03/PC04 and GH20/PC04 primer sets (10). HPV screening was done by the nested PCR procedure using MY09-MY11 as outer and GP5+-GP6+ as inner primers as previously described (11).

**Statistical Considerations**

The data was analyzed using SPSS 13.0[Statistical Procedures for Social Sciences; Chicago, Illinois, USA]. The Fisher test was used and was considered statistically significant at P-values of less than 0.05.

## Results

Cancer patients had a mean age of 48.2±10.8 years. The types and percentage of malignant tumors are summarized in Table 1. About 49.3% of cancers were in the right breast and 50.7% in the left side. The mean age in the control group was 38.9 years and 76% of lesions were right-sided. The benign lesions were FCC, FCC with sclerosing adenosis, and FCC with focal adenomatoid change (57.3%), fatty breast tissue (25.3%), ductal ectasia (1.3%), and other types (fat necrosis and reduction mastectomy specimens, 16%). HPV DNA was not identified in any of the malignant or control group specimens.

**Table1:** Types of malignant tumors in current study

Type	Percentage
**Invasive ductal carcinoma (IDC)**	88.01
**Invasive lobular carcinoma (ILC)**	9.33
**Tubular carcinoma**	1.33
**Medullary carcinoma**	1.33
**Total**	100

## Discussion

 Tryouts to avoid virus-related malignancies would be complicated but not unattainable (2). Viruses such as HPV, *EBV*, and *MMTV* are the major candidates of hormone-responsive viruses; they are believed to cause breast cancer in humans (4, 7, 8). In the current study, we failed to detect HPV DNA in any of the malignant or control groups, suggesting no etiological association between breast carcinoma and HPV in our patients. 

No connection is revealed between viruses and breast malignancies in some studies, while others suggest possible etiological role for viruses. The incidence of HPV infection in patients with breast malignancy in different countries is between zero in Brazil, Germany, London and France to 16% in Mexico and Greece, 46.5% in Iraq, between 20.9% and 86.2% in Australia, Syrian and Turkey (12), and 47% in the United Kingdom (6). The incidence of malignant breast lesions having HR-HPV has been reported between 0-2% in various regions of China to 86% among North Americans (12, 13). 

Lindel *et al.* (14) found no HPV DNA in 81 tissues from breast carcinoma. Tsai *et al*. (3) used southern hybridization and PCR methods in 122 patients with malignant and benign breast lesions. HPV was seen in 12.9% and in 6.2% of malignant and benign lesions respectively, demonstrating no significant difference. The research by Joshi and colleagues (15) showed no definite connecting relationship between carcinoma of breast and HPV. Liang *et al*. (16) also reported no significant difference among 224 breast cancers and 37 fibroadenomas using Hybrid capture assay (HPV was detected in 21.4% and 16.2% in breast cancer and fibroadenoma groups, respectively). HPV DNA was not discovered in 130 benign and malignant breast tissues, employinga PCR technique by Romano and colleagues (17). Choi and his team (18), using real-time PCR, identified HPV in 17.9% of 123 cancers and 22.2% of nine intraductal papillomas. These patients were confirmed to have HPV infection of uterine cervix. Nevertheless; the findings were not supportive of any connection between breast malignancy and HPV due to the weak positive results.

Contrary to the above studies, in Antonsson’s study (19) 54 cases with breast cancer were assessed and showed 50% positivity for HPV DNA (in 27 patients) using In Situ Hybridization (ISH). All cases were HPV-18. Glenn *et al.* (20) reported 50% and 20% positivity for HPV DNA in breast cancer and control groups, showing significant difference. Lower age and higher grade were related with a positive result for HPV. Salman’s (6) screening of 110 fresh breast tissue specimens for HR-HPV DNA detected positivity in 31%of the benign breast tissue and 47% of breast carcinoma cases, and suggested that virus-related oncoprotein selective appearance in infiltrative tumors can be suggestive of HR-HPVs role in the growth of a number of tumors in breast. Suarez and colleagues (21) evaluated 61 breast cancers and found 26% positive cases for HPV DNA. They suggested possible etiological function. Suarez’s 26% positivity for HPV DNA in 61 breast carcinomas implied a possible etiological role. The result of a meta-analysis by Bae *et al.* (22) on22 case-control investigations was in favor of the hypothesis of HPV infection raising the possibility of cancer in breast tissue. The review article by Hsu *et al.* (4) revealed important connection between breast malignancy and HPV and showed a relationship between cervical and breast cancer, demonstrating a common etiological function of HPV. 

The studies that have been carried out in Iran (on 1678 malignant and benign breast tissues; 1828 samples from Iranian women, including our cases) are demonstrated in Table 2. All studies were done by PCR and the ones that showed a possible positive association of HPV DNA with breast cancer are: Sigaroudi *et al*. (23) with a 25.9% positive rate for HPV in cancerous breasts and a predominance of high-risk types in the north of Iran, Alavi and colleagues (24) with 48% positivity in Mahad (east of Iran), Ghaffari *et al.* (25) with 30% positivity in Tehran (occurrence rate of HPV16, 11 ⁄ 6, and 31 ⁄ 33 were 14.9%, 11.94%, and 2.99% respectively) and Rassi (26) with 34.66% HPV positivity in Tehran and Karaj, central north of Iran (the predominant genotype was the HPV16 and 18). Other studies from different parts of Iran that did not show any association include: Isfahan, midwest of Iran (27), Tehran, north of Iran (28), Tabriz, west of Iran (29), Golestan, northeast of Iran (30), Roudeh Hen, north of Tehran (31), Yazd, central part of Iran (32), Sanandaj, west of Iran (33) and Mashhad, east of Iran (34). 

**Table 2:** Results of studies in Iran

Study	Case Group	Control Group	Method	Association of high-risk HPV with breast cancer
**Ahangar et al. (** 29 **)**	22/65 (33.8%)	0/65	Nested PCR	No (low-risk HPV detected)
**Sigaroudi et al. (** 23 **)**	16/79 (25.9%)	1/51	Nested PCR	Yes (predominantly high-risk types)
**Manzouri et al. (** 27 **)**	10/55 (18.18%)	7/51(12.72%)	PCR	No
**Alavi et al. (** 24 **)**	24/50 (48%)	0/29	PCR	Yes
**Rassi et al. (** 26 **)**	52/150 (34.66%)	------	Multiplex PCR	Yes (types16 and 18)
**Salehpour et al. (** 34 **)**	54/206 (26.2%)	-------	PCR	No (low-risk HPV detected)
**Doosti et al. (** 32 **)**	20/87 (22.9%)	0/84	Nested PCR	No (low-risk HPV detected)
**Tahmasebi Fard (** 31 **)**	0/65	3/53	PCR	No
**Moradi et al. (** 30 **)**	0/231	-----	PCR	No
**Eslamifar et al. (** 28 **)**	0/100	0/50	Nested PCR	No
**Ghaffari et al. (** 25 **)**	20/67 (30%)	-	PCR	Yes (types 16, 11 ⁄ 6, and 31 ⁄ 33)
**Karimi et al. (** 33 **)**	2/70	0/70	PCR	No
**Current study**	0/75	0/75	PCR	No
**Total**	200/1300 (15.3%)	11/528 (2.08%)	----	

Some investigators have found that the genotype of HPV determines its pathogenicity and the high-risk ones (HPV 16, 18 and 33) are to blame for 70% of breast carcinomas worldwide (4, 7). The reported HPV types in Iran include 16, 11 ⁄ 6, and 31 ⁄ 33 (25), and 16 an 18 (26); however, in the cancerous cases in the United Kingdom the most common types were 39,18,45,16,35, and 59 (6), and in India, the most common reported HPV types in malignant lesions were 16, 18 and 33 (9).

Such inconsistency in the results obtained from various regions in Iran could be attributed to different genotypes of HPV and the incidence of high-risk behavior. Other issues which can affect the results include storage condition of the specimens, quality of samples used, and the quality of PCR procedure (12). 

According to Haghshenas *et al.* (12) that carried out a meta-analysis on the abovementioned studies examining the incidence of HPV infection in cancerous breast lesions of Iranian female patients, the combined incidence rate (95% confidence interval) of infection by HPV was 23.6% (6.7- 40.5), and the odds ratio (95% confidence interval) between breast cancer development and HPV infection was 5.7% (0.7- 46.8). This analysis demonstrated a high incidence of this viral infection in Iranian patients with breast malignancy.

Although our results showed no HPV DNA in malignant or benign breast tissues, the review of Iranian literatures is in favor of a role for this virus in growth of breast cancer in Iranian patients. Diverse geographic distributions of virus, different assessment techniques, and diverse subtypes could be suggested as the main cause of difference between results obtained in various studies worldwide.
